# Understanding the impact of antibiotic therapies on the respiratory tract resistome: a novel pooled-template metagenomic sequencing strategy

**DOI:** 10.1186/s40248-018-0140-9

**Published:** 2018-08-09

**Authors:** Steven L. Taylor, Lex E. X. Leong, Fredrick M. Mobegi, Jocelyn M. Choo, Lucy D. Burr, Steve Wesselingh, Geraint B. Rogers

**Affiliations:** 1 Infection and Immunity, South Australian Health and Medical Research Institute, Adelaide, South Australia Australia; 2 SAHMRI Microbiome Research Laboratory, College of Medicine and Public Health, Flinders University, Adelaide, South Australia Australia; 3 Department of Respiratory Medicine, Mater Health Services, South Brisbane, Queensland, Australia

**Keywords:** Antibiotic therapy, DNA, Metagenomic screening

## Abstract

Determining the effects of antimicrobial therapies on airway microbiology at a population-level is essential. Such analysis allows, for example, surveillance of antibiotic-induced changes in pathogen prevalence, the emergence and spread of antibiotic resistance, and the transmission of multi-resistant organisms. However, current analytical strategies for understanding these processes are limited. Culture- and PCR-based assays for specific microbes require the a priori selection of targets, while antibiotic sensitivity testing typically provides no insight into either the molecular basis of resistance, or the carriage of resistance determinants by the wider commensal microbiota. Shotgun metagenomic sequencing provides an alternative approach that allows the microbial composition of clinical samples to be described in detail, including the prevalence of resistance genes and virulence traits. While highly informative, the application of metagenomics to large patient cohorts can be prohibitively expensive. Using sputum samples from a randomised placebo-controlled trial of erythromycin in adults with bronchiectasis, we describe a novel, cost-effective strategy for screening patient cohorts for changes in resistance gene prevalence. By combining metagenomic screening of pooled DNA extracts with validatory quantitative PCR-based analysis of candidate markers in individual samples, we identify population-level changes in the relative abundance of specific macrolide resistance genes. This approach has the potential to provide an important adjunct to current analytical strategies, particularly within the context of antimicrobial clinical trials.

**Electronic supplementary material:**

The online version of this article (10.1186/s40248-018-0140-9) contains supplementary material, which is available to authorized users.

## Introduction

As in all clinical disciplines, the management of patients with chronic respiratory diseases is subject to a process of ongoing refinement, including through the development of novel antimicrobial drugs and treatment strategies. Understanding the impact of antimicrobial treatments for individual recipients allows the personalisation of clinical management. However, determining the effects of treatments at a population level is also crucial, providing a means to predict shifts in the prevalence of respiratory pathogens, or the emergence of antimicrobial resistance, within large patient groups.

The impact that evolving treatment strategies can have on airway microbiology can be seen, for example, in changes in the cystic fibrosis (CF) airway microbiota during recent decades. Within this context, the use of anti-Pseudomonal treatments, including parenteral therapies and fluoroquinolones, have been implicated in the emergence of *Stenotrophomonas maltophilia* as an airway pathogen [1, 2]. Likewise, increasingly intensive antibiotic use appears to be a contributory factor in the increasing prevalence of non-tuberculous mycobacteria [3, 4]. The impact of antibiotic use is also reflected in the increasing frequency of multi-drug resistant organisms in the airways of patients with chronic respiratory disease, with an estimated 25–45% of adult CF patients chronically infected with multi-drug resistant bacteria [5]. For example, CF-derived methicillin-resistant *Staphylococcus aureus* (MRSA) isolates increasingly show resistance to newer therapies, including linezolid [6, 7], ceftaroline [8] and tigecycline [6], presumably as a result of frequent and prolonged exposures [9].

Despite the importance of understanding the impact of antimicrobial exposure on the airway microbiome in those with respiratory disease, characterising this process remains challenging. Assessments of antibiotic-associated changes in microbiology are typically limited to a small group of predefined pathogens or resistance genes. The standard analytical approaches employed in clinical antibiotic trials fail to assess major aspects of antibiotic resistance, including shifts in the composition of the wider airway microbiota, and the carriage of transmissible resistance determinants by populations of commensal microbes. The absence of suitable strategies to determine antibiotic impact has resulted in significant gaps in our understanding of how widely employed therapies affect the complex microbiota of the respiratory tract.

Shotgun metagenomic sequencing is an emerging technology that allows highly detailed characterisation of airway microbiota through the analysis of total microbial DNA from clinical samples, including determination of the prevalence of virulence factors and resistance determinants [10]. While metagenomic approaches have been shown to be highly effective in describing changes in the microbiome across a wide range of clinical contexts [11], the cost of its employment within population-scale studies is commonly prohibitive.

We describe a novel, cost-effective, strategy to inform the use of assays for specific resistance genes or microbial taxa, based on deep metagenomic screening of pooled study cohort DNA. We illustrate the application of this approach through the analysis of samples from a previously reported randomised controlled trial of long-term low dose macrolide therapy in adults with bronchiectasis.

## Methods

The BLESS randomised placebo-controlled trial assessed the effect of 12 months of low dose erythromycin therapy (twice-daily erythromycin ethylsuccinate; 400 mg) on exacerbation rates in adults with non-CF bronchiectasis [12]. The analysis reported here was based on paired baseline and week 48 sputum samples from 32 members of the treatment group, and subsequent analysis between treatment group and placebo group subjects (*n* = 32, and *n* = 31, respectively). Patient baseline characteristics are described in Additional file 1: Table S1.

Sputum DNA extracts were pooled according to time-point and subject to microbial DNA enrichment (NEBNext® Microbiome DNA Enrichment Kit). DNA fragmentation was performed with TruSeq Nano DNA Library Prep Kit (Illumina), prior to 150 bp paired-end metagenomic shotgun sequencing using the Illumina HiSeq system at the South Australian Health and Medical Research Institute, Adelaide. Reads have been uploaded to the Sequence Read Archive (SRA) under BioProject ID: 397083.

Sequences were quality filtered using Trimmomatic v0.32 [13] and human-derived reads removed using BBMap v35.40 (comparing reads to the NCBI human reference genome release GRCh38) [14]. Contigs were de novo assembled using IDBA-UD v1.1.1 [15], followed by identification of open-reading frames using MetaGeneMark v3.26 [16]. A non-redundant gene catalogue was constructed using CDHIT v4.6 [17] and resistome composition annotated by BLASTP search to the Comprehensive Antibiotic Resistance Database (CARD) v1.1.7 [18]. Quantification of gene hits was determined by SOAP v2.20 [19] and normalised to counts per million reads.

Specific resistance genes that were identified as associated with erythromycin treatment through metagenomics were subsequently quantified in DNA extracts from individual sputum samples by qPCR. Previously published assays were used for *ermA* [20]*, ermB* [21], *ermC* [22]*,* 16S [23], and *smpB* [24] genes. Primers for quantification of *hmrM* were designed within this study (see Additional file 1). For analysis of qPCR results, Wilcoxon rank tests were performed on fold change normalised to 16S copy number to compare erythromycin paired samples to placebo control paired samples (*n* = 31 pairs).

## Results

**Fig. 1:**
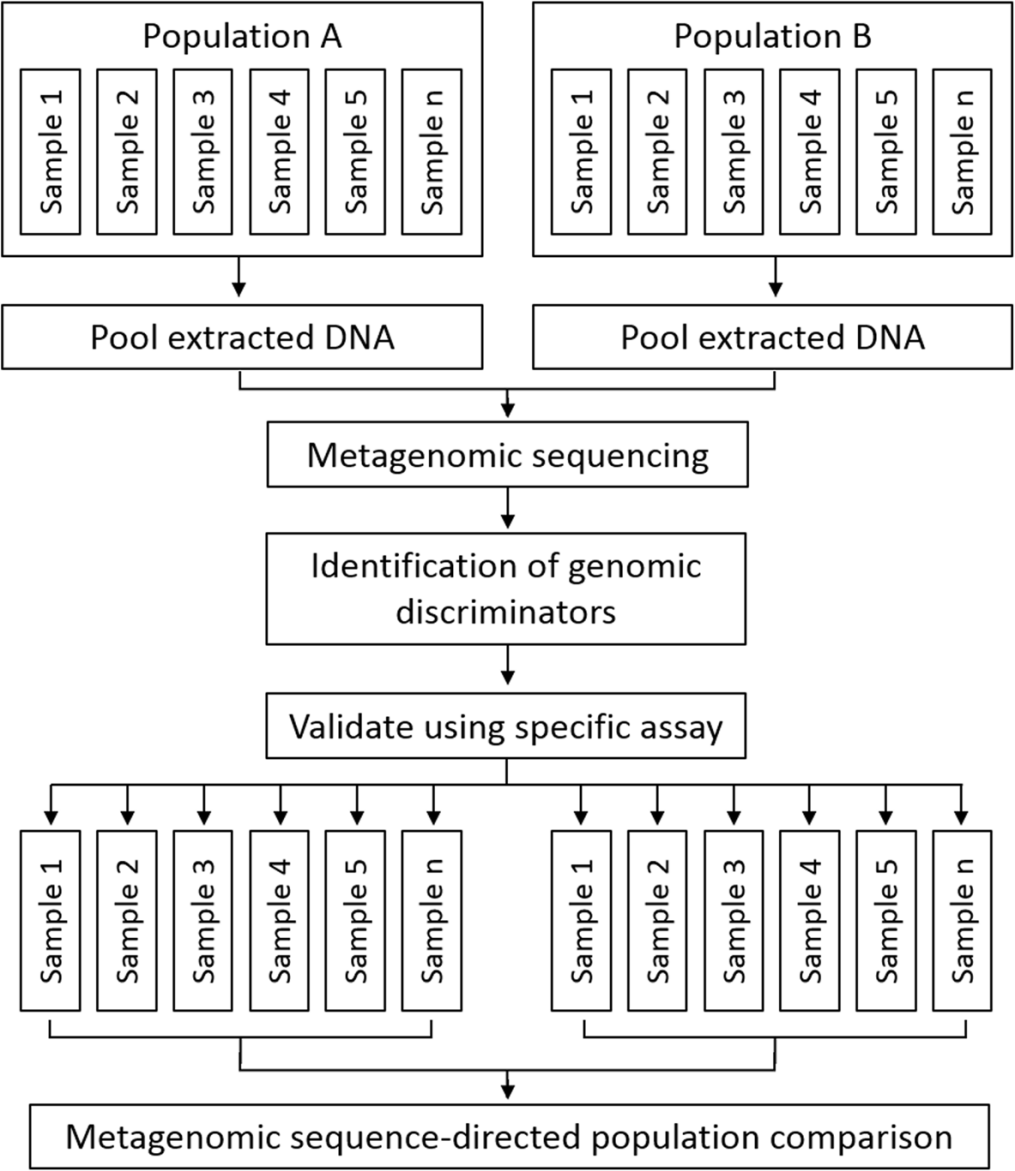
Principle of pooled-template metagenomic sequencing. Sample DNA extracts from a population of interest are pooled together according to a pre-specified variable of interest (such as treatment or time-point). Metagenomic sequencing is then performed on pooled samples and regions that discriminate between populations are determined. Targeted assays (such as qPCR) are then performed on individual samples for gene specific enumeration

**Fig. 2:**
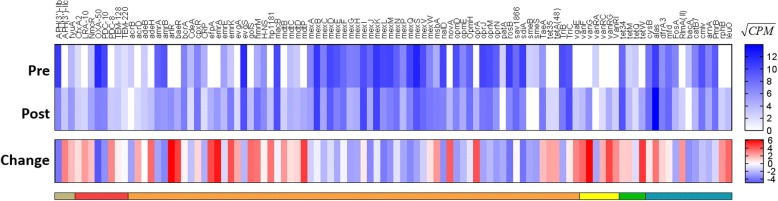
Resistome of pooled-template sputum before and after erythromycin therapy. Square root-transformed counts per million total reads (CPM) of major antibiotic resistance genes identified by CARD database. Change in CPM where red indicates higher in samples post erythromycin. Resistance genes grouped by function as defined by CARD where: brown = aminoglycoside resistance genes, red = beta-lactam resistance genes, orange = efflux pump resistance genes, yellow = glycopeptide resistance genes, green = tetracycline resistance genes, blue = other resistance genes

A schematic of the pooled-template metagenomic sequencing strategy, and subsequent qPCR-based validation, is shown in Fig. 1. Following removal of low-quality reads and human DNA (approximately 90% of total read depth), a mean sample read depth of 12,866,780 was achieved. Approximately half a million reads has been previously found to be sufficient for sputum metagenome analysis in individual samples [25]. Mapping of sequence reads to the CARD database resulted in the detection of a total of 102 resistance-associated genetic determinants. The distribution of normalised reads that mapped to the CARD database in pre- and post-trial pooled samples is shown in Fig. 2. Detected genes represented a range of resistance mechanisms, including antibiotic inactivating enzymes, efflux pumps, and effector site protection proteins, and conferred resistance to a number of antibiotic classes, including aminoglycosides, beta-lactams, glycopeptides, and tetracyclines.

**Fig. 3:**
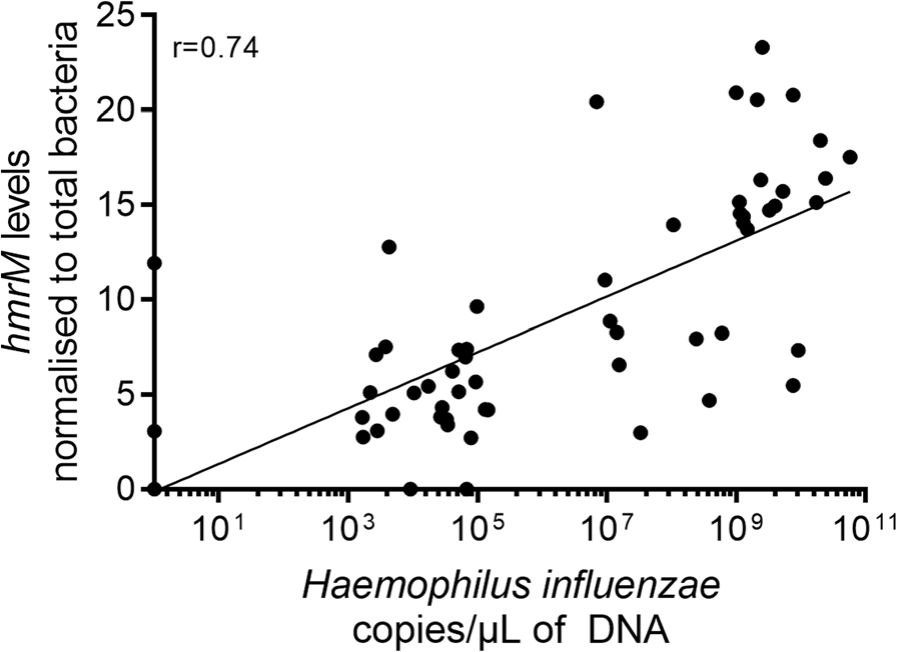
Correlation between *hmrM* and *H. influenzae* copy number. *hmrM* (normalised to total bacteria) against *H. influenzae* copy number (determined by comparing to known standard curve). Significance determined by Spearman’s rank order correlation

A substantial proportion of the genes identified through resistome analysis were chromosomally-encoded, non-transmissible, resistance determinants. Changes in the level of carriage of these genes during the trial therefore reflected shifts in the relative abundance of the species in whose genomes they are encoded, rather than resistance gene acquisition or loss. For example, the multidrug efflux pump gene, *hmrM*, appeared to increase in response to erythromycin therapy. This gene is chromosomally-encoded by *Haemophilus influenzae* however, and subsequent qPCR analysis revealed *hmrM* levels to be correlated with *H. influenzae* levels (*r* = 0.74, *p* < 0.001, Fig. 3). The observed increase in prevalence of *hmrM* is therefore likely to simply reflect an increase in the relative abundance in *H. influenzae* in the assessed patient group (a median increase of 1.4 × 10^3^ copies was observed between pre- and post-erythromycin samples). This phenomenon could explain apparent changes in the group-level abundance of other chromosomally-encoded resistance genes, such as an observed decrease in the relative abundance of *patA*, a chromosomally-encoded fluoroquinolone resistance gene carried by *Streptococcus pneumoniae* [26], and *aph(3′)-IIb*, a chromosomally-encoded aminoglycoside resistance gene carried by *Pseudomonas aeruginosa* [27].

**Fig. 4:**
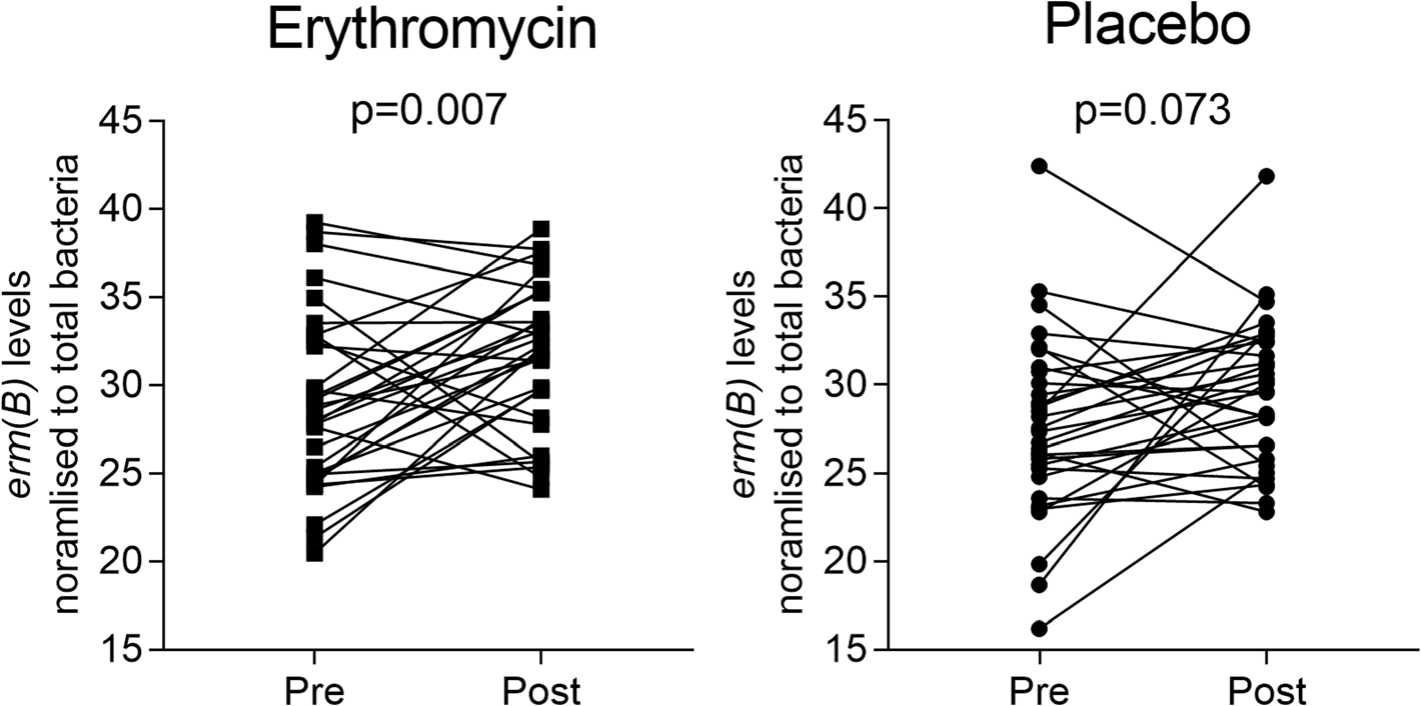
Changes in *ermB* levels in erythromycin and placebo groups. Paired sample analysis of *ermB* (normalised to total bacteria). Significance determined by Wilcoxon signed-rank test

Many of the other resistance genes identified through pooled-template metagenomic sequencing were, however, encoded on mobile genetic elements, and have been shown previously to be transmissible between bacterial species. These include a number of transmissible genetic elements that confer resistance to macrolide antibiotics. For example, qPCR-based validation analysis, revealed a significant increase in the relative abundance of the plasmid-encoded erythromycin resistance methylase gene, *ermB*, in subjects who received erythromycin (*p* = 0.007), but not in those who received placebo (*p* = 0.073, Fig. 4). The *ermB* gene can be carried by a number of respiratory pathogens, including *S. pneumoniae*, *S. aureus*, and *H. influenzae* [28–30], and confers substantial resistance to all macrolide drugs. In contrast, other transmissible macrolide-resistance determinants were shown by follow-up qPCR analysis to not contribute substantially to the post-trial resistome. For example, *ermA*, a resistance gene found in staphylococci [29], was present in only four subjects (two in the treatment group and two in the control group). The *ermC* resistance determinant, which is also found in staphylococci [29], was detected more frequently (68% of subjects receiving placebo and 81% of subjects receiving erythromycin), however, *ermC* levels did not change significantly over the course of the trial. The rates of carriage of *ermA* and *ermC* are consistent with those reported in *S. aureus* clinical isolates more widely [29, 31].

## Discussion and conclusion

We describe a cost effective approach that can be used to guide the assessment of changes in antibiotic resistance gene carriage, which might represent a useful adjunct to conventional approaches that are based on a priori target selection. As an illustration, the BLESS randomised placebo-controlled trial that preceded this study included an assessment of whether erythromycin therapy resulted in an increased relative abundance of macrolide resistant oropharyngeal streptococci using culture-based proportional sensitivity testing [12]. While this narrow analysis identified a significant increase in the proportion of macrolide-resistant streptococci, neither the level of transmissible resistance gene carriage in non-streptococcal species, nor the molecular basis of resistance, were determined. Our use of pooled metagenomic sequencing revealed a number of resistance determinants for follow-up analysis where targeted qPCR assays were subsequently applied to DNA extracts from individual samples. This validation step confirmed significant increases in the abundance of, for example, the transmissible macrolide resistance gene, *ermB*, in patients receiving erythromycin.

By pooling sample DNA at the pre-sequencing, rather the post-sequencing, library-construction stage (as performed in standard metagenomic sequencing approaches), we calculate the cost of our analysis to be approximately 15% of that required to analyse all of the samples individually (although precise costs will be influenced by sample number, processing methodologies, and desired sequencing depth). However, despite this substantial reduction in expense, it is important to be aware of some of the limitations that are inherent in this approach. For example, variations in bacterial load between samples from different patients mean that pooling DNA based on total concentration could result in the contribution of individual samples to meta-microbiome characteristics being unequal. In addition, the non-normal distribution of microbiome traits within a population could lead to the identification of traits as potential inter-group discriminators based on their particularly high abundance in a small number of individuals (although the impact of this effect is likely to decrease with increasing cohort size).

A limitation of all metagenomic sequencing is the challenge to differentiate between changes in the carriage of resistance determinants due to direct selective pressure versus changes in resistance gene carriage, because of shifts in the relative abundance of the bacterial populations that encode them. Due to such limitations, the approach that we describe should be used as an additional means to identify markers for further analysis, rather than as a means to characterise antibiotic associated effects on airway microbiology in itself.

As an illustration of the potential of the pooled-template metagenomic analysis, we examined shifts in the airway resistome. This application targeted the global health concern of monitoring of antibiotic resistance. Patients with chronic lung diseases have an increased exposure to antibiotics, with the emergence of resistance correlating closely with consumption [32]. The resistome associated with the airway microbiota in these patients is likely to be a substantial contributor to the emergence and expansion of populations of multi-resistant organisms [33] and their potential transmission to individuals within the wider community. However, despite its application to the assessment of the airway resistome here, pooled-template metagenomic analysis can be applied equally to assessment of species distribution [34], or to identify changes in community level carriage of pathogenicity traits (for example, through alignment to virulence factor genetic databases). By aligning regions that encode antibiotic binding sites, it may also be possible to determine the relative abundance of resistance-conferring single nucleotide polymorphisms (SNPs). Obtaining such information could provide important clinical insight. For example, while de novo mutations in the 23S rRNA are the principal cause of macrolide resistance in non-tuberculous mycobacteria [35], relatively little is known currently about the dynamics of their emergence during macrolide therapy.

The costs of metagenomic sequencing, and the associated costs of high performance computing required for bioinformatic analysis, are likely to continue to fall. However, by providing a low-cost means to perform unbiased surveys of large patient cohorts, strategies such as the one described here represent a useful means of identifying potentially important discriminatory microbiome features for follow-up analysis.

## Additional file

Additional file 1: hmrM primers and patient characteristics. (DOCX 18 kb)
