# Supplementary material for: Understanding the impact of antibiotic therapies on the respiratory tract resistome: a novel pooled-template metagenomic sequencing strategy
Source: Multidiscip Respir Med. 2018 Aug 9;13(Suppl 1):30. doi: 10.1186/s40248-018-0140-9 (PMC6101085; doi:10.1186/s40248-018-0140-9)
Supplement: Supplementary file 1 — hmrM primers and patient characteristics. (DOCX 18 kb) [file 40248_2018_140_MOESM1_ESM.docx]

**Additional file 1**

**hmrM primer design**

Designed using NCBI Primer BLAST and validated by performing qPCR on *H. influenzae* clinical isolate and non-*Haemophilus* controls.

Forward primer: GTGGAGAACCTGCACCCAAT
Reverse primer: AATTTGTTGCGAAGTGGCGT

Product length: 182 bp

Thermocycling conditions: 95°C for 30 s, 60°C for 60 s

**Table S1**

|  | Erythromycin (n=32) | Placebo (n=31) |
| --- | --- | --- |
| Age, mean yrs (std) | 63.5 (7.9) | 64.1 (9.9) |
| Gender, female (%) | 19 (59.4) | 15 (48.4) |
| Duration of Bronchiectasis, mean yrs (std) | 45.3 (20.2) | 41.4 (23.3) |
| Ex-smoker, n (%) | 8 (25) | 7 (22.6) |
| Inhaled corticosteroids, n (%) | 5 (15.6) | 4 (12.9) |
| PDPE, median (IQR) | 1 (0-2) | 2 (1-3) |
| FEV_1_/FVC, mean (std) | 0.676 (0.09) | 0.701 (0.09) |
| FEV_1_ absolute, mean (std) | 1.77 (0.56) | 1.83 (0.78) |
| FEV_1_ % predicted, mean (std) | 65.6 (16.6) | 68.3 (21.4) |
| PDPE: physician defined pulmonary exacerbation  Std: standard deviation  IQR: interquartile range | | |
